# A national sample of developmentally disabled adolescents with obesity and their utilization of preventive dental care services

**DOI:** 10.3389/froh.2023.1285416

**Published:** 2023-11-09

**Authors:** Nini C. Tran, Christine R. Wells, Kathryn A. Atchison, Vinodh Bhoopathi

**Affiliations:** ^1^ Section of Pediatric Dentistry, School of Dentistry, University of California, Los Angeles, Los Angeles, CA, United States; ^2^ Statistical Methods and Data Analytics, Office of Advanced Research Computing, University of California, Los Angeles, Los Angeles, CA, United States; ^3^ Section of Public & Population Health, School of Dentistry, University of California, Los Angeles, Los Angeles, CA, United States

**Keywords:** developmental disabilities, adolescents, obesity, dental sealants, fluoride treatment, dental cleanings, dental visits

## Abstract

**Background:**

Previous literature indicates that adolescents with developmental disabilities and obesity may have more oral health complications than healthy adolescents. However, dental care utilization among adolescents with developmental disabilities (DDs) and obesity is unclear. We investigated the differences in the utilization of preventive dental services between this high-risk group of adolescents and those with no DDs or obesity.

**Methods:**

Parent-reported data of adolescents 10–17 years (*n* = 68,942) from the 2016 to 2019 National Survey of Children's Health was used. In addition to descriptive and bivariate statistics, we ran three multiple logistic regression models guided by Andersen's Behavioral Model of Health Services Use, predicting the use of dental cleanings, fluoride treatments, and dental sealants.

**Results:**

Among adolescents with DDs and obesity, dental cleanings, fluoride treatments, and dental sealant utilization prevalence were 76%, 48%, and 21%, respectively. In comparison, adolescents with no DDs or obesity had a prevalence of 83%, 50%, and 19%, respectively. Multiple logistic regression analysis showed that adolescents with DDs and obesity did not significantly differ in their receipt of dental cleanings (*p = .*07), fluoride treatments (*p = .*55), and dental sealants (*p = .*23) compared to those with neither DDs nor obesity. Adolescents with DDs but no obesity were 22% and 30% more likely to receive fluoride treatments (*p *< .0001) and dental sealants (*p *< .0001), respectively.

**Conclusions:**

Fewer than half of adolescents with DDs and obesity utilized fluoride treatments, and less than one quarter utilized dental sealants but used all three preventive services at the same rate as those with no DDs or obesity.

**Implications:**

This study identified no differences in preventive dental care services utilization in adolescents with developmental disabilities (DDs) and obesity compared to those without DDs and obesity. However, the utilization of preventive dental services in this population is influenced by the federal poverty level and family background.

## Introduction

Almost two decades ago, dental caries was described in the first-ever U.S. Surgeon General's report as the most common childhood disease (1). In the recent National Institute of Dental and Craniofacial Research report released in 2021, dental caries was still reported as one of the most common childhood diseases (2). National level data (2011–2016) show that dental caries prevalence is highest in 12 to 19-year-old adolescents (57%), followed by 2 to 5-year-olds (23%), and 6 to 11-year-olds (17%) (3).

Childhood obesity is a substantial public health crisis in the U.S. (4). In 2017–2018, 12 to 19-year-old adolescents had a higher obesity prevalence (21%) compared to 3 to 5-year-olds (13%) and 6 to 11-year-olds (20%) (4). A 2017 scoping review showed that obesity and dental caries share non-modifiable and modifiable common risk factors (5). Developmental conditions, a subset of conditions under special health care needs, were identified as a common risk factor for both these diseases (5).

Children and adolescents with developmental disabilities (DDs) experience complex lifelong health issues (6, 7). Prevalence of DDs between 2015 and 2017 was highest for 12 to 17-year-old adolescents (21%), then for 6 to 11-year-olds (18%) and 3 to 5-year-olds (11%) (8). The obesity risk among DD adolescents 10 to 17 is significantly higher than among adolescents without DDs (9). The higher obesity risk among adolescents with DDs may be associated with ailments that limit their access to physical activities and proper nutrition (9). This indicates adolescents with DDs and obesity may be high-risk and have high health care needs, including oral health care needs.

It is recommended that individuals prioritize their health by seeking preventive healthcare services, including dental care (10). Fluoride treatments and dental sealants are non-invasive preventative treatments that can effectively reduce dental caries in children and adolescents (11). These preventive dental care services may further reduce the need for non-preventive dental visits (12, 13). However, according to Andersen's Behavioral Model of Health Services Use, the utilization of such services is often influenced by predisposing, enabling, and need-related factors (14).

Adolescents with developmental disabilities may experience difficulties maintaining regular dental routines due to cognitive and behavioral challenges, potentially negatively impacting their oral health (15). Therefore, preventive dental services are vital to maintaining optimal oral health in these adolescents. One study of Medicaid youth aged 3–17 years showed a significantly lower proportion (36.3%) of children with Autism Spectrum Disorders (ASDs) utilized preventive dental care than children without ASDs (45.7%) (16). However, in another study of Medicaid children and adolescents ages 3–17 years with intellectual developmental disabilities (IDD) were as likely to receive preventive dental care services as those without IDD (17). While these studies provide some insights into how children and adolescents with a specific type of DDs utilize dental care, currently, there is a lack of national-level studies describing the association between preventive dental care service utilization among adolescents impacted by DDs and obesity, a high-risk group of adolescents. Therefore, using national-level data, we first assessed the prevalence of preventive dental care services utilization among adolescents with DDs and obesity and then evaluated differences in preventive dental care services utilization between adolescents with DDs and obesity compared to adolescents with no DDs or obesity controlling for predisposing, enabling, and need factors.

## Methods

### Data source, study design, and participants

The National Survey of Children Health (NSCH) survey collects cross-sectional representative data from parents or guardians (hereafter called parents) of children aged 0–17 years. The Maternal Child Health Bureau sponsors it. A screener questionnaire was used during data collection to identify households with children and roster children in the household. Questions to identify special health care needs were included in the screener questionnaire. From each eligible household, one child who was the subject of a detailed topical questionnaire was randomly selected.

The Centers for Disease Control and Prevention define children ages 10–19 as adolescents (18). For this analysis, we used parent-reported data of adolescents 10–17 years from 2016 through 2019 NSCH surveys. We used Andersen's Behavioral Model of Health Services Use to guide our research (14).

### Study variables

#### Outcome variables

To evaluate preventive dental services, we analyzed parent-reported data on their child's visits to a dentist or other dental care provider for preventive dental care in the past 12 months and whether specific preventive services were received during those visits. We examined three commonly prescribed services: dental cleaning, fluoride treatments, and dental sealants.

#### Main independent variable

A primary independent variable (Developmental disability and obesity status) was created to categorize adolescents into four groups: (a) with DDs and obesity, (b) with DDs but no obesity, (c) with obesity but no DDs, and (d) no DDs or obesity. This variable was created from two other variables, one that identified the adolescents with DDs vs. no DDs, and the other with obesity/overweight vs. no obesity/overweight. A previously validated case definition by the Centers for Disease Control and Prevention (CDC) researchers and Health Resource Services Administration (HRSA) was used to identify adolescents with and without DDs (8). An adolescent was considered to have one or more DDs if the adolescent had any of the following: ASDs, attention deficit hyperactivity disorder (ADHD), blindness, cerebral palsy, hearing loss/deafness, learning disability, intellectual disability, seizures in the past 12 months, stuttering or stammering in the past 12 months, or any other developmental delay. Because the “any other developmental delay” option was available only in the 2016–2018 data, we analyzed these cycles to determine if estimates changed drastically without 2019 data. However, the models and associations remained the same when the data for all four years (2016–2019) was combined for analysis. Following the CDC's definition of weight status, we categorized adolescents as having a weight problem (obese or overweight) when their Body Mass Index (BMI) was in the 85th percentile and above and not obese or overweight when the BMI was below the 85th percentile (19).

#### Predisposing variables

Demographic characteristics and social conditions are the predisposing factors that may influence a person's decision to use a preventive dental service (14, 20). The following predisposing factors to utilizing preventive dental care services were used: age (10–13 years/14–17 years), gender (male/female), race/ethnicity of the adolescent (Non-Hispanic White/Non-Hispanic Black/Hispanics/Other), and social factors like highest education of household (less than high school/high school or GED/some college or technical school/college degree or higher), and language spoken in the home (English/Spanish/Other).

#### Enabling variables

Income or dental insurance gives children regular access to a dentist (14, 20). We included the Family Poverty Level (FPL) variable (0%–199%, 200%–400%, 400%, and more) to represent income and the insurance variable (public only/private only/private and public/insurance type unspecified/not insured) as the key enabling factors.

#### Need variables

Factors representing the potential need for preventive dental care service use include dental diseases or other conditions (14, 20). Parents were asked, “During the past 12 months, has this child had frequent or chronic difficulty with any of the following?”. Of those listed, the following health complications associated with oral health were used for this analysis: (a) toothaches, (b) decayed teeth or cavities, (c) bleeding gums, and (d) eating or swallowing because of a health condition. Adolescents were categorized as having zero oral health complications, one or two oral health complications, or three or more oral health complications. Developmental disability and obesity status, the main independent variable described above, is also considered a need variable.

### Data analysis

NSCH-recommended weighting procedures were applied to account for non-response, complex sampling, and survey design. Weighted prevalence estimates of the outcome, primary independent, predisposing, enabling, and need variables were derived. Bivariate associations between main independent, predisposing, enabling, and need variables with the three outcome variables (dental cleaning, fluoride treatments, dental sealants) were tested using *χ*^2^ tests. To determine the differences in preventive dental care service utilization between adolescents with DDs and obesity and those without DDs or obesity, we conducted three multiple adjusted logistic regression models controlling for predisposing, enabling, and need factors. Listwise deletion was used to handle missing data, except for the FPL variable. The FPL variable had six multiple imputation replicates because of missing data and was used in the analyses. Stata version 17 was used to conduct all data analytical procedures. The statistical significance was set at *p *< 0.05.

## Results

The analytical sample comprised 68,942 adolescents aged 10–17 years. Table 1 shows the weighted characteristics of the overall analytical sample and the sample stratified by the four categories of the DD and obesity status variable. Males (51%) represented slightly more than half of the sample and were over-represented in DDs and obesity (61%) and DDs with no obesity (62%) groups. Almost 51% of the sample was Non-Hispanic White, who also comprised the majority in all the 4 DD and obesity categories compared to other racial/ethnic groups. Most adolescents were from English-speaking families (86%), and approximately half (46%) were from families where the highest level of education attained was a college degree or higher. Similar scenarios were seen across all 4 DD and obesity groups by language spoken and the highest level of education in the household. Almost 52% of adolescents with no DDs or obesity belonged to households led by an adult with a college degree or above, compared to only 31% of those families of adolescents with both DDs and obesity. Forty-two percent were in the lowest FPL category, and 31% were in the more than 400% FPL category. Among those below 200% FPL, 54% had both DDs and obesity, compared with 35% of the adolescents who neither had a DD nor obesity among those in the more than 400% FPL category. Similarly, while most adolescents had private-only insurance (59%), 29% had public-only insurance; however, 65% of those with private insurance had no DD or obesity compared to 23% among the public-only insurance families. Almost 85% of the sample had no oral complications, with a much higher proportion of those without DDs and obesity (87%) having no oral complications compared to only 76% of those with DDs and obesity having no oral complications. Nearly 82%, 50%, and 20% of adolescents had received dental cleanings, fluoride treatments, and dental sealants, respectively, in the past 12 months. Only 76% of those with DDs and obesity received dental cleanings compared to those with DDs only (83%) or those with neither DDs nor obesity (83%). Most adolescents had no DDs or obesity (57%), 22% had obesity but no DDs, 14% had DDs but no obesity, and 7% had both DDs and obesity.

**Table 1:** Analytical sample characteristics stratified by developmental disability and obesity status.

	Overall (*n* = 68,942)	DDs and obesity	DDs but no obesity	Obesity without DDs	No DDs or obesity	*p*-value^a^
	(%)	(%)	(%)	(%)	(%)	
Age						
10–13	50%	54%	51%	54%	48%	**<**.**001**
14–17	50%	46%	49%	46%	52%	
Gender						
Male	51%	61%	62%	52%	47%	**<**.**001**
Female	49%	39%	38%	48%	53%	
Race/ethnicity						
Hispanic	25%	27%	22%	32%	23%	**<**.**001**
Non-Hispanic White	51%	48%	57%	41%	53%	
Non-Hispanic Black	14%	18%	13%	18%	13%	
Other	10%	7%	8%	9%	11%	
Language spoken in house						
English	86%	89%	90%	82%	85%	**<**.**001**
Spanish	10%	9%	8%	15%	10%	
Other	4%	2%	2%	3%	5%	
Highest Education level in household						
Less than high school education	11%	14%	10%	15%	10%	**<**.**001**
High school or GED^b^ diploma	20%	27%	20%	25%	17%	
Some college or technical school	23%	28%	23%	25%	21%	
College degree or higher	46%	31%	47%	35%	52%	
Federal poverty level						
0–199%	42%	54%	45%	49%	37%	**<**.**001**
200–400%	27%	26%	24%	28%	28%	
More than 400%	31%	20%	31%	23%	35%	
Type of insurance coverage						
Public only	29%	43%	34%	36%	23%	**<**.**001**
Private only	59%	40%	52%	51%	65%	
Private and Public	4.6%	11%	7%	4%	4%	
Insurance type not specified	0.4%	1%	1%	1%	1%	
Not insured	7.0%	5%	6%	8%	7%	
Frequent or chronic difficulty in the last 12 months due to one or more oral health complications						
No complications	85%	76%	79%	85%	87%	**<**.**001**
1 complication	11%	17%	14%	12%	10%	
2 complications	3%	5%	6%	2%	2%	
3 or more complications	1%	2%	1%	1%	1%	
Received dental cleanings						
Yes	82%	76%	83%	79%	83%	
No	18%	24%	17%	21%	17%	**<**.**001**
Received fluoride treatments						
Yes	50%	48%	55%	46%	50%	
No	50%	52%	45%	54%	50%	**<**.**001**
Received dental sealants						
Yes	20%	21%	24%	19%	19%	
No	80%	79%	76%	81%	81%	**<**.**001**
Developmental disabilities (DDs) and Obesity status						
With DDs but no obesity	14%					
With obesity but no DDs	22%					
No DDs nor obesity	57%					
With both DDs and obesity	7%					

^a^ Bold indicates *p* value <0.05.

^b^ GED, Graduate Education Development.

Results from logistic regression analyses (Table 2) showed that adolescents with both DDs and obesity, compared to those with neither DDs or obesity, had a similar receipt of dental cleanings (*p = *.07), fluoride treatments (*p = *.55), and dental sealants (*p = *.23). A similar association was observed between adolescents with obesity but no DDs and those with no DDs or obesity. Adolescents with DDs but no obesity were significantly more likely to receive fluoride treatments (OR: 1.22, 95% CI: 1.09–1.36) and dental sealants (OR: 1.30, 95% CI: 1.14–1.48) compared to those with no DDs or obesity.

**Table 2:** Logistic regression models: characteristics of adolescents who utilized preventive dental care services in the past 12 months.

	Dental Cleaning		Fluoride Treatment		Sealants	
	Adj OR (95% CI)	*p*-value^a^	Adj OR (95% CI)	*p*-value^a^	Adj OR (95% CI)	*p*-value^a^
Developmental disabilities (DDs) and obesity						
With DDs but no obesity	1.06 (0.90–1.25)	.48	1.22 (1.09–1.36)	**<** **.** **001**	1.30 (1.14–1.48)	**<** **.** **001**
With obesity but no DDs	.95 (0.82–1.09)	.48	0.95 (0.87–1.05)	.33	1.00 (0.89–1.13)	.93
With both DDs and obesity	0.81 (0.65–1.01)	.07	1.04 (0.90–1.22)	.55	1.11 (0.93–1.32)	.23
No DDs nor obesity	[Reference]		[Reference]		[Reference]	
Age						
10–13	1.27 (1.14–1.42)	**<** **.** **001**	1.61 (1.49–1.73)	**<** **.** **001**	1.60 (1.46–1.74)	**<** **.** **001**
14–17	[Reference]		[Reference]		[Reference]	
Gender						
Male	0.87 (0.78–0.98)	**.** **02**	0.99 (0.92–1.07)	.82	0.90 (0.82–0.99)	**.** **02**
Female	[Reference]		[Reference]		[Reference]	
Race/ethnicity						
Hispanic	0.79 (0.66–0.95)	**.** **02**	0.83 (0.74–0.95)	.004	1.12 (0.95–1.31)	.17
Non-Hispanic Black	0.59 (0.51–0.68)	**<** **.** **001**	0.61 (0.55–0.69)	<.001	0.87 (0.75–1.00)	.06
Other	0.86 (0.72–1.01)	.07	0.74 (0.66–0.82)	<.001	1.02 (0.90–1.15)	.72
Non-Hispanic White	[Reference]		[Reference]		[Reference]	
Language spoken in house						
Spanish	1.03 (0.78–1.35)	.85	0.54 (0.43–0.68)	**<** **.** **001**	0.61 (0.44–0.85)	**.** **004**
Other	0.53 (0.40–0.68)	**<** **.** **001**	0.44 (0.35–0.55)	**<** **.** **001**	0.53 (0.38–0.73)	**<** **.** **001**
English	[Reference]		[Reference]		[Reference]	
Highest Education level in household						
Less than high school education	0.45 (0.36–0.58)	**<** **.** **001**	0.49 (0.40–0.61)	**<** **.** **001**	0.55 (0.42–0.74)	**<** **.** **001**
High school or GED^b^ diploma	0.48 (0.42–0.56)	**<** **.** **001**	0.58 (0.52–0.64)	**<** **.** **001**	0.79 (0.68–0.91)	**.** **002**
Some college or technical school	0.60 (0.52–0.68)	**<** **.** **001**	0.73 (0.67–0.80)	**<** **.** **001**	0.88 (0.79–0.94)	**.** **01**
College degree or higher	[Reference]		[Reference]		[Reference]	
Federal poverty level						
0–199%	0.51 (0.43–0.61)	**<** **.** **001**	0.71 (0.64–0.79)	**<** **.** **001**	0.81 (0.71–0.92)	**.** **009**
200–400%	0.63 (0.54–0.73)	**<** **.** **001**	0.86 (0.79–0.93)	**.** **005**	0.99 (0.89–1.11)	.92
More than 400%	[Reference]		[Reference]		[Reference]	
Type of insurance coverage						
Public only	0.91 (0.77–1.08)	.30	0.91 (0.81–1.02)	.12	1.13 (0.99–1.29)	.07
Private and Public	1.05 (0.78–1.41)	.76	0.90 (0.74–1.10)	.32	1.18 (0.92–1.52)	.19
Insurance type not specified	0.68 (0.35–1.30)	.24	0.62 (0.38–1.02)	.06	0.61 (0.32–1.14)	.12
Not insured	0.43 (0.34–0.53)	**<** **.** **001**	0.43 (0.36–0.52)	**<** **.** **001**	0.80 (0.60–1.06)	.12
Private only	[Reference]		[Reference]		[Reference]	
Frequent or chronic difficulty in the last 12 months due to one or more oral health complications						
1 complication	1.19 (1.00–1.42)	.05	1.11 (0.98–1.28)	.11	1.52 (1.29–1.78)	**<** **.** **001**
2 or more complications	0.86 (0.65–1.14)	.31	0.96 (0.78–1.18)	.72	1.35 (1.05–1.75)	**.** **02**
No complications	[Reference]		[Reference]		[Reference]	

^a^ Bold indicates *p* value <0.05.

^b^ GED, Graduate Education Development.

The following are some notable findings (Table 2). Hispanic adolescents, compared to non-Hispanic White adolescents, were significantly at lower odds of receiving dental cleanings (*p *= 0.02) and fluoride treatments (*p *= .004) but not dental sealants (*p *= 0.17). Likewise, non-Hispanic Black adolescents, compared to non-Hispanic White adolescents, were significantly at lower odds of receiving dental cleanings (*p *< .001) and fluoride treatments (*p *< .001), but not dental sealants. Adolescents from other language-speaking households, compared to those from English-speaking families, were at lower odds of receiving dental cleanings *(p *< .001), fluoride treatments *(p *< .001), and dental sealants *(p *< .001). In comparison, adolescents from Spanish-speaking households were at lower odds of receiving fluoride treatments *(p *< .001) and dental sealants *(p *< .001) but not dental cleanings (*p *= 0.85). Household education level and FPL were significantly associated with receiving three preventive dental care services. Those from lower FPL and lower education level households consistently received preventive dental care services at a lower rate than families with over 400% FPL and families with college degree and higher.

## Discussion

A higher prevalence of DDs, obesity, and dental caries is observed in adolescents than in their younger counterparts (3, 8, 9). A recent study demonstrated that adolescents with DDs and obesity experience chronic difficulty with many oral health complications at a rate significantly higher than those without DDs or obesity (21). In that study, authors found that adolescents with both DDs and obesity were significantly more likely to have experienced difficulty with dental caries in the past 12 months compared to those without DDs or obesity (21). Therefore, these high-risk adolescents must receive dental care services regularly to prevent oral health complications. We, therefore, investigated the differences in preventive dental services utilization among adolescents with DDs and obesity vs. those with no DDs or obesity. Logistic regression analysis showed that adolescents with DDs and obesity received dental cleanings, fluoride treatments, and dental sealants at the same rate as those without DDs or obesity. So did adolescents with obesity but without DDs. However, adolescents with only DDs were the only group that had utilized preventive dental services more than those with no DDs or obesity. Parents of DD adolescents may perceive a greater need for dental care and, therefore, take their children to a dentist. Additionally, the data shows that a higher proportion of adolescents with only DDs but no obesity had private only insurance (52%), were from a household with the highest level of education (college degree or higher) (47%), and English-speaking households (90%). The data showed that adolescents with both DDs and obesity did not receive preventive services at a higher rate than those with no DDs or obesity despite a majority of the adolescents from this group belonging to two high-risk subgroups: below 200% FPL (54%) and with public insurance (43%).

Parents reported that almost 4 in 5 adolescents had received dental cleanings in the past 12 months. However, fluoride treatments and dental sealants were reported by only 50% and 20%, respectively. The American Dental Association (ADA) (22) and the American Academy of Pediatric Dentistry (AAPD) (23) specifically recommend professionally applied fluoride treatments for children and adolescents with high caries risk. A lower proportion of children receiving fluoride treatments could mean that adolescents in this study were at a low risk of dental caries. It may also suggest that the high rate of dental cleaning may have put the adolescents at a low caries risk. Alternatively, it is possible that the use of fluoride treatments may be limited by insurance benefit coverage, or parents may not recall their child receiving the treatment. Applying sealants on sound occlusal surfaces and non-cavitated occlusal carious lesions of primary and permanent molars in children and adolescents is highly recommended (24). However, there are various reasons for the lower prevalence of dental sealant. One possible explanation is that parents may not recall the event or procedure of sealant placement, which is recommended to be done as soon as the first molar erupts at an early age of 6–7 years old (24). Another possibility is that parents may not fully understand what sealants are.

In this study, adolescents from a household that spoke a non-English language (NEL) were primarily less likely to receive preventive dental services (except dental cleanings in Spanish-speaking families) than those from English-speaking households. Previous literature shows some contradicting results. One group of researchers indicates that those from NEL-speaking households have reduced odds of preventive dental visits compared to English-speaking families (25). Another group of researchers reported no association between the language spoken and the utilization of dental services (26). Studies show that NEL-speaking parents of children have difficulty getting insured due to language barriers, misinformation, and lack of knowledge of insurance programs (27). This lack of health insurance coverage could indicate that the adolescent may be unable to visit a dentist and, therefore, be unable to utilize dental care services. Our research findings indicate that adolescents covered by public insurance are as likely to receive all three preventive dental care services as their counterparts covered solely by private insurance. This promising observation may suggest equitable access to dental care services for all individuals, regardless of their insurance coverage.

Dental sealant utilization prevalence was low among all subgroups, including those from different racial/ethnic groups. There was no difference in dental sealant utilization by race/ethnicity. However, non-Hispanic Black and Hispanic adolescents consistently utilized dental cleanings and fluoride treatments at a much lower rate than non-Hispanic White adolescents suggesting racial and ethnic disparities in using specific dental services among adolescents.

The study has limitations due to parental recall of their child's health status and experiences. Parent reports were not confirmed clinically in NSCH, so there is a chance of underestimation or overestimation of actual results. Non-response bias is also possible, like any other survey; however, no strong evidence of non-response bias has been identified in NSCH. Despite these limitations, this is the first study to have investigated the utilization of preventive dental care services among a very high-risk group of adolescents with DDs and obesity.

## Conclusions

The study detected a low prevalence of fluoride treatments and dental sealant utilization in adolescents with DDs and obesity. However, no significant differences in receipt of the three preventive services were observed between adolescents with a DD and obesity and adolescents with no DDs or obesity. Federal Poverty Level and family background play an important role in adolescents' utilization of preventive dental services.

## Data Availability

Publicly available datasets were analyzed in this study. This data can be found here: https://www.childhealthdata.org/Learn-about-the-nsch/NSCH
